# Liver fibrosis indices associated with substantial hematoma expansion in Chinese patients with primary intracerebral hemorrhage

**DOI:** 10.1186/s12883-021-02494-0

**Published:** 2021-12-09

**Authors:** Huan Wang, Jiongxing Wu, Xue Yang, Junfeng Liu, Wendan Tao, Zilong Hao, Bo Wu, Ming Liu, Shihong Zhang, Deren Wang

**Affiliations:** Department of Neurology, Center of Cerebrovascular Disease, West China Hospital, Sichuan University, No. 37 Guo Xue Xiang, Chengdu, 610041 Sichuan Province People’s Republic of China

**Keywords:** Hemorrhagic stroke, Liver fibrosis indices, Hematoma enlargement, AST–platelet ratio index, Fibrosis-4

## Abstract

**Background:**

Whether liver fibrosis is associated with increased risk for substantial hematoma expansion (HE) after intracerebral hemorrhage (ICH) is still uncertain. We evaluated the association between various liver fibrosis indices and substantial HE in a Chinese population with primary ICH.

**Methods:**

Primary ICH patients admitted to West China Hospital within 24 h of onset between January 2015 and June 2018 were consecutively enrolled. Six liver fibrosis indices were calculated, including aspartate aminotransferase (AST)-platelet ratio index (APRI), AST/alanine aminotransferase ratio-platelet ratio index (AARPRI), fibrosis-4 (FIB-4), modified fibrosis-4 (mFIB-4), fibrosis quotient (FibroQ) and Forns index. Substantial HE was defined as an increase of more than 33% or 6 mL from baseline ICH volume. The association of each fibrosis index with substantial HE was analyzed using binary logistic regression.

**Results:**

Of 436 patients enrolled, about 85% showed largely normal results on standard hepatic assays and coagulation parameters. Substantial HE occurred in 115 (26.4%) patients. After adjustment, AARPRI (OR 1.26, 95% CI 1.00-1.57) and FIB-4 (OR 1.15, 95% CI 1.02-1.30) were independently associated with substantial HE in ICH patients within 24 h of onset, respectively. In ICH patients within 6 h of onset, each of the following indices was independently associated with substantial HE: APRI (OR 2.64, 95% CI 1.30-5,36), AARPRI (OR 1.55, 95% CI 1.09-2.21), FIB-4 (OR 1.35, 95% CI 1.08-1.68), mFIB-4 (OR 1.09, 95% CI 1.01-1.18), FibroQ (OR 1.08, 95% CI 1.00-1.16) and Forns index (OR 1.37, 95% CI 1.10-1.69).

**Conclusions:**

Liver fibrosis indices are independently associated with higher risk of substantial HE in Chinese patients with primary ICH, which suggesting that subclinical liver fibrosis could be routinely assessed in such patients to identify those at high risk of substantial HE.

**Supplementary Information:**

The online version contains supplementary material available at 10.1186/s12883-021-02494-0.

## Introduction

Intracerebral hemorrhage (ICH) accounts for 10% to 15% of all strokes, and causes up to 50% of all stroke-related mortality [1–3]. More than one-third of patients with ICH experience hematoma expansion (HE), which is a strong predictor of early deterioration and death [4–6]. To date, randomized controlled trials of several interventions targeting HE have not demonstrated any improvement in outcomes after ICH [7–9]. Therefore, understanding the risk factors of HE in patients with ICH may help to guide preventive measures and intervention post-ICH.

Patients with unusually large hematomas after ICH show elevated levels of aspartate aminotransferase (AST) and alanine aminotransferase (ALT) [10]. ICH patients with comorbid liver cirrhosis are more likely to die in-hospital and are in worse condition at discharge [11]. Even subclinical liver disease may be associated with worse outcome post-ICH [12]. Liver fibrosis is a common subclinical liver disease that is present in approximately 15% of the general adult population without diagnosed liver disease [13, 14]. Several noninvasive markers have been reported to predict liver fibrosis: AST/ALT ratio (AAR), AST–platelet ratio index (APRI), AAR/platelet ratio index (AARPRI), fibrosis-4 (FIB-4), modified fibrosis-4 (mFIB-4), fibrosis quotient (FibroQ) and the Forns index [15–17]. These liver fibrosis indices allow simple, inexpensive, straightforward assessment of liver fibrosis, including in patients with viral hepatitis, non-alcoholic fatty liver disease (NAFLD) and alcohol-related liver disease (ALD) [15–21].

In addition, a recent study in patients with largely normal liver enzyme levels in a predominantly Caucasian population found that liver fibrosis indices were associated with outcomes post-ICH [22]. However, there is little evidence on the relationship between liver fibrosis indices and HE in Asian patients without obvious liver diseases. Therefore, we aimed to evaluate the association between liver fibrosis indices and substantial HE after primary ICH in a Chinese population.

## Materials and methods

### Study design and cohort selection

Patients with primary ICH who were admitted to West China Hospital within 24 h of ICH symptom onset between January 2015 and June 2018 were retrospectively selected from among patients prospectively registered in our ICH database [23]. The study was approved with waived consent by the Ethics Committee on Biomedical Research, West China Hospital of Sichuan University (2019 362).

Patients were included if they received a baseline brain computed tomography (CT) scan immediately upon arrival in the Department of Emergency Medicine, as well as a follow-up brain CT scan within 72 h after symptom onset. Patients were excluded if they met any of the following criteria: (1) were < 18 years old; (2) had a secondary cause of ICH (i.e., underlying aneurysm, vascular malformation, brain neoplasm or metastasis, head trauma, dural sinus thrombosis, hemorrhagic transformation of ischemic infarction, infection), or primary intraventricular hemorrhage (IVH), or primary subarachnoid hemorrhage (SAH); (3) were on anticoagulant therapy before admission; (4) had severe liver disease (i.e., an ICD-code for liver failure, cirrhosis, hepatocellular carcinoma or decompensated liver disease) or clinical syndrome associated with liver disease; (5) had undergone surgical evacuation; or (6) were lacking laboratory data of hepatic assays or coagulation parameters, which were tested routinely in most of patients.

### Data collection

The following data were collected for all patients: demographic information including age, sex, Glasgow Coma Scale (GCS) score, National Institutes of Health Stroke Scale (NIHSS) score, as well as systolic and diastolic blood pressure on admission; comorbidities diagnosed either before admission or at discharge (hypertension, diabetes mellitus, hyperlipidemia, heart disease (includes any history of atrial fibrillation, coronary heart disease, or valvular heart disease); previous smoking (defined as smoking more than 10 cigarettes a day prior to onset, for at least 1 year) and alcohol consumption (defined as drinking almost every day, the average amount of daily drinking being more than 50 g, for more than 1 year); and the use of antiplatelets before admission. In addition, data were collected on the previous history of stroke (ischemic or hemorrhagic stroke).

Laboratory findings on admission included platelet count, international normalized ratio (INR), prothrombin time (PT), activated plasma thromboplastin time (APTT), ALT, AST and albumin (ALB). Data were also collected about onset of ICH symptoms, how long the patient was symptom-free, and when baseline and follow-up brain CT scans were performed.

The initial CT scan taken at presentation was reviewed to determine the ICH location (lobar, deep, and infratentorial), the presence of IVH, and baseline hematoma volumes. Hematoma volumes were estimated from CT scans using the formula A*B*C/2, where A was the largest diameter on the largest hemorrhage slice; B, the maximal diameter perpendicular to A; and C, the vertical hematoma depth [24]. Intraventricular hemorrhage were not considered for volumetric calculations.

### Estimation of liver fibrosis indices

We selected six liver fibrosis indices: APRI, AARPRI, FIB-4, mFIB-4, FibroQ, and Forns index. Fibrosis indices were calculated according to the following formulas [16, 17, 21]:

$$\mathrm{APRI}=\frac{\mathrm{AST}\ \left(/\mathrm{ULN}\right)}{\mathrm{Platelet}\ \mathrm{count}\ \left({10}^9/\mathrm{L}\right)}\times 100.$$

$$\mathrm{AAR}=\frac{\mathrm{AST}}{\mathrm{ALT}}\mathrm{ratio},\mathrm{AARPRI}=\frac{\mathrm{AAR}}{\mathrm{Platelet}\ \mathrm{count}\ \left({10}^9/\mathrm{L}\right)/150}$$

$$\mathrm{FIB}-4=\frac{\mathrm{Age}\ \left(\mathrm{years}\right)\times \mathrm{AST}\left(\mathrm{U}/\mathrm{L}\right)}{\mathrm{Platelet}\ \mathrm{count}\ \left({10}^9/\mathrm{L}\right)\times \sqrt{\mathrm{ALT}\left(\mathrm{U}/\mathrm{L}\right)}}$$

$$\mathrm{mFIB}-4=\frac{10\times \mathrm{Age}\ \left(\mathrm{years}\right)\times \mathrm{AST}\ \left(\mathrm{U}/\mathrm{L}\right)}{\mathrm{Platelet}\ \mathrm{count}\ \left({10}^9/\mathrm{L}\right)\times \mathrm{ALT}\ \left(\mathrm{U}/\mathrm{L}\right)}$$

$$\mathrm{FibroQ}=10\times \frac{\mathrm{Age}\ \left(\mathrm{years}\right)\times \mathrm{AST}\left(\mathrm{U}/\mathrm{L}\right)\times \mathrm{INR}}{\mathrm{ALT}\left(\mathrm{U}/\mathrm{L}\right)\times \mathrm{Platelet}\ \mathrm{count}\ \left({10}^9/\mathrm{L}\right)}$$

$$\mathrm{Forns}\ \mathrm{index}=7.811-3.131\times \mathrm{In}\ \left(\mathrm{Platelet}\ \mathrm{count}\left[{10}^9/\mathrm{L}\right]\right)+0.781\times \mathrm{In}\ \left(\upgamma \mathrm{GT}\ \left[\mathrm{IU}/\mathrm{L}\right]\right)+3.467\times \mathrm{In}\ \left(\mathrm{age}\right)-0.014\times \mathrm{cholesterol}\ \left(\mathrm{mg}/\mathrm{dl}\right)$$

### Outcome

The outcome of interest was substantial HE within 72 h of symptom onset. Substantial HE was defined as a proportional increase of hematoma volume>33% or an absolute growth of hematoma volume>6 mL from baseline CT scan to follow-up CT scan [25].

### Statistical analysis

Categorical variables were presented as n (%), whereas continuous variables were presented as mean ± standard deviation (SD) or median and interquartile range (IQR). Differences between groups with or without substantial HE were assessed for significance using the chi-squared test or Fisher exact test in the case of categorical variables, or using Student’s t test or Mann-Whitney U test in the case of continuous variables.

Potential associations between liver fibrosis indices and substantial HE were estimated using binary logistic regression. All variables associated with *p* ≤ 0.10 were considered in the regression. Laboratory data were not included as variables in the regression, since the liver fibrosis indices were estimated from such data. To avoid effects of interactions between liver fibrosis indices, each index was included separately in the binary logistic regression. Since hematoma volume on admission may affect the association between liver fibrosis indices and substantial HE, we considered two models: Model 1 and Model 2, which contained the same variables as Model 1 in addition to baseline hematoma volume. Analysis was planned both in all patients and an early admission group within 6 h of ICH onset.

Statistical analyses were conducted using SPSS 24.0 (IBM, New York, USA). Differences and statistical results associated with a 2-tailed p<0.05 were defined as significant.

## Results

### Baseline patient characteristics

**Fig. 1:**
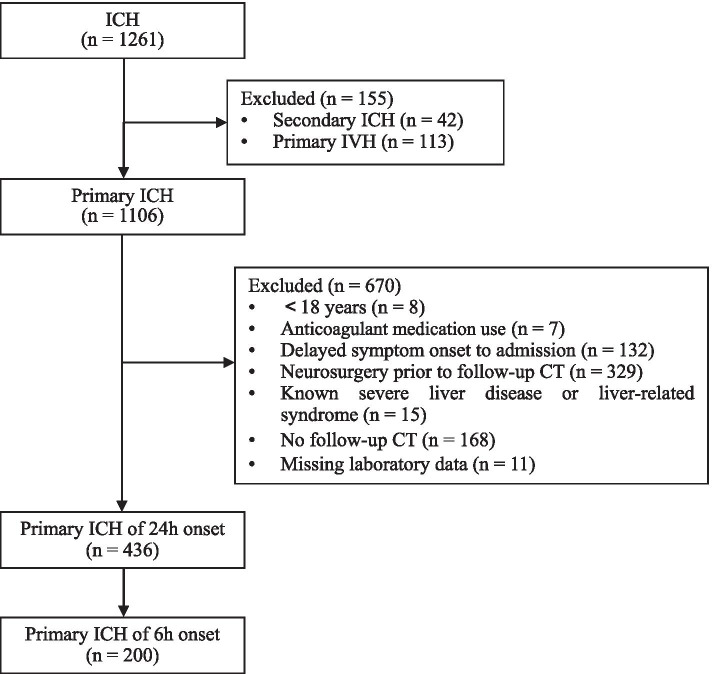
Selection of the study cohort

We identified 1261 patients from the ICH database between January 2015 and June 2018. There were 1106 patients with primary ICH, of whom 436 patients were within 24 h of symptom onset and finally included. Of the 436 patients, 200 patients were within 6 h of symptom onset. A flowchart depicting patient selection is shown in Fig. 1.

The characteristics of included patients (*n* = 436) and excluded (*n* = 670) patients in our registry are shown in Table 1. The patients included in this study were older (p<0.001), and had a higher prevalence of diabetes mellitus (*p* = 0.026), better baseline clinical severity scores (GCS and ICH score, all p<0.001), higher antiplatelet medication use (*p* = 0.008) and faster times from symptom onset to admission CT (*p* < 0.001). In addition, there were smaller baseline hematoma volumes (p < 0.001), a lower prevalence of IVH (*p* = 0.036) and a greater proportion of deep ICH (p < 0.001) in included patients. Finally, the included patients had shorter PT (*p* = 0.019) and lower INR (*p* = 0.002).

**Table 1:** Patient characteristics stratified by study inclusion

Characteristic	Included (n = 436)	Excluded (n = 670)	*p* Value	OR (95% CI)
Age, years	59.9 (14.0)	55.1 (15.1)	<0.001	NA
Male	292 (67.0)	428 (63.9)	0.292	1.147 (0.889-1.479)
GCS	14 (9-15)	12 (6-14)	<0.001	NA
NIHSS	9 (3-14)	11 (4-27)	<0.001	NA
BP on admission, mmHg				NA
Systolic BP	166.2 (26.9)	162.7 (32.8)	0.053	
Diastolic BP	95.8 (16.8)	97.0 (22.4)	0.296	
Comorbidities				
Hypertension	275 (63.1)	395 (59.0)	0.171	1.189 (0.928-1.524)
Diabetes mellitus	40 (9.2)	38 (5.7)	0.026	1.680 (1.059-2.665)
Dyslipidemia	120 (27.5)	177 (26.4)	0.685	1.058 (0.806-1.388)
Heart disease	28 (6.4)	28(4.2)	0.096	1.574 (0.919-2.696)
Stroke history	44 (10.1)	52 (7.8)	0.179	1.334 (0.876-2.032)
Smoking	124 (28.4)	188 (28.1)	0.891	1.019 (0.780-1.332)
Alcohol consumption	125 (28.7)	172 (25.7)	0.272	1.164 (0.888-1.525)
HBV infection	302/436 (69.3)	451/629 (71.7)	0.391	0.889 (0.681-1.162)
Antithrombotic medication				
Antiplatelet drugs	12 (2.8)	5 (0.7)	0.008	3.764 (1.317-10.760)
Anticoagulants	0 (0)	7 (1.0)	0.047	NA
ICH volume, ml	15.5 (7.6-33.5)	28.3 (10.1-49.8)	<0.001	NA
Location of bleed			<0.001	NA
Deep location	287 (65.8)	319 (47.6)		
Lobar location	95 (21.8)	213 (31.8)		
Infratentorial location	54 (12.4)	138 (20.6)		
IVH	124 (28.4)	231 (34.5)	0.036	0.755 (0.581-0.982)
Time from ICH onset to baseline scan, h	6.5 (4.0-12.5)	9.5 (5.0-25.0)	<0.001	NA
Laboratory findings				NA
Platelet count, × 10^9^/L	158 (121-201)	164 (123-213)	0.141	
International normalized ratio	1.0 (0.9-1.0)	1.0 (0.9-1.1)	0.002	
Prothrombin time, sec	11.4 (10.9-12.0)	11.6 (11.0-12.3)	0.019	
APTT, sec	26.5 (24.3-29.3)	26.6 (24.0-29.4)	0.871	
Aspartate transaminase, IU/L	24 (20-31)	24 (19-31)	0.391	
Alanine transaminase, IU/L	21 (15-30)	20 (14-29)	0.511	
Albumin, g/dL	43.4 (41.2-45.8)	43.0 (40.2-45.6)	0.026	

Values are n (%), mean (SD) or median (interquartile range)

*Abbreviations*: *GCS* Glasgow Coma Scale, *NIHSS* National Institutes of Health Stroke Scale, *BP* blood pressure, *HBV* hepatitis B virus, *CT* computed tomography, *ICH* intracerebral hemorrhage, *IVH* intraventricular hemorrhage, *APTT* activated partial thromboplastin time

The included patients were predominately male (67.0%) and had a mean age of 59.9 years (± 14.0 years), with ages ranging from 20 to 94 years (Table 1). As expected, about 85% of patients had generally normal standard liver chemistry examination indices and coagulation indices; 12.6% had an AST ≥ 40 IU/L, 8.9% had an ALT ≥50 IU/L, and 6.9% had an INR ≥ 1.15. Moreover, the abnormal values of ALT, AST or INR were within two times the upper limit of the normal value (ULN).

### Association between liver fibrosis indices and substantial HE in patients within 24 h of ICH onset

Of 436 patients within 24 h of ICH onset, 115 (26.4%) had substantial HE (Table 2). In general, patients with substantial HE were older (mean 63.0 vs 58.8 years, *p* = 0.007), tended to be male (74.8% vs 64.2%, *p* = 0.038), had larger baseline hematoma volumes (median 24.3 vs 13.0 ml, *p* = 0.005) and a lower proportion of deep ICH (52.2% vs 70.7%, *p* < 0.001). The baseline GCS score, blood pressure on admission, the time from symptom onset to baseline CT and the time from baseline CT to follow-up CT scan were similar between groups (all p>0.05).

**Table 2:** Characteristics of ICH patients within 24 h of ICH symptom onset (n = 436), stratified by HE

Characteristic	With HE (*n* = 115)	Without HE (*n* = 321)	p Value	OR (95% CI)
Age, years	63.0 (14.5)	58.8 (13.7)	0.007	NA
Male	86 (74.8)	206 (64.2)	0.038	1.656 (1.026-2.672)
GCS	13 (9-15)	14 (9-15)	0.766	NA
NIHSS	9 (3-14)	8 (3-14)	0.773	NA
BP on admission, mmHg				NA
Systolic BP	165.4 (27.5)	166.6 (26.8)	0.680	
Diastolic BP	94.5 (18.1)	96.3 (16.4)	0.323	
Comorbidities				
Hypertension	76 (66.1)	199 (62.0)	0.435	1.195 (0.764-1.868)
Diabetes mellitus	13 (11.3)	27 (8.4)	0.356	1.388 (0.690-2.792)
Dyslipidemia	27 (23.5)	93 (29.0)	0.258	0.752 (0.459-1.233)
Heart disease	8 (7.0)	20 (6.2)	0.785	1.125 (0.481-2.630)
Stroke history	13 (11.3)	31 (9.7)	0.615	1.192 (0.601-2.367)
Smoking	39 (33.9)	85 (26.5)	0.129	1.425 (0.900-2.254)
Alcohol consumption	35 (30.4)	90 (28.0)	0.626	1.123 (0.705-1.789)
HBV infection	80 (69.6)	222 (69.2)	0.935	1.019 (0.642-1.619)
Antiplatelet drugs	4 (3.5)	8 (2.5)	0.579	1.410 (0.416-4.774)
ICH volume, ml				NA
At admission	24.3 (8.3-45.4)	13.0 (7.6-30.5)	0.005	
At follow-up	33.8 (16.5-56.2)	13.2 (6.7-30.0)	<0.001	
Location of bleed			0.001	NA
Deep location	60 (52.2)	227 (70.7)		
Lobar location	37 (32.2)	58 (18.1)		
Infratentorial location	18 (15.7)	36 (11.2)		
IVH	29 (25.2)	95 (29.6)	0.372	0.902 (0.494-1.302)
Symptom onset to first CT, h	6.0 (3.5-10.5)	7.0 (4.0-13.0)	0.083	NA
Time between the first and second CT, h	22.0 (4.0-32.0)	23.0 (7.0-38.0)	0.340	NA
Laboratory findings				NA
Platelet count, ×10^9^/L	140 (112-202)	160 (124-200)	0.038	
International normalized ratio	1.0 (0.9-1.0)	1.0 (0.9-1.0)	0.887	
Prothrombin time, sec	11.4 (10.8-12.1)	11.4 (10.9-12.0)	0.788	
APTT, sec	26.5 (24.3-29.3)	26.5 (24.3-29.3)	0.844	
Aspartate transaminase, IU/L	25 (20-32)	24 (20-31)	0.652	
Alanine transaminase, IU/L	20 (15-28)	21 (15-32)	0.518	
Albumin, g/dL	43.3 (41.2-46.1)	43.4 (41.1-43.4)	0.835	
Liver fibrosis index				NA
APRI	0.4 (0.3-0.7)	0.4 (0.3-0.6)	0.245	
AARPRI	1.3 (0.8-1.8)	1.1 (0.8-1.6)	0.069	
FIB 4	2.4 (1.6-3.9)	2.1 (1.3-3.0)	0.016	
mFIB 4	5.2 (3.4-8.5)	4.4 (2.6-7.1)	0.015	
FibroQ	5.2 (3.4-8.1)	4.3 (2.6-7.1)	0.024	
Forns index	6.6 (5.2-7.9)	6.2 (4.9-7.3)	0.017	

Values are n (%), mean (SD) or median (interquartile range)

*Abbreviations*: *APRI* AST to platelet ratio index, *AARPRI* AAR/platelet ratio index, where AAR is aspartate aminotransferase/alanine aminotransferase (AST/ALT) ratio, *FIB-4* fibrosis-4, *mFIB-4* modified fibrosis-4, *FibroQ* fibrosis quotient, *GCS* Glasgow Coma Scale, *NIHSS* National Institutes of Health Stroke Scale, *BP* blood pressure, *HBV* hepatitis B virus, *CT* computed tomography, *HE* hematoma expansion, *ICH* intracerebral hemorrhage, *IVH* intraventricular hemorrhage, *APTT* activated partial thromboplastin time

Patients with substantial HE showed significantly better values for AARPRI, FIB-4, mFIB-4, FibroQ and Forns index than those without substantial HE (all p<0.1), but there was no statistically significant difference in APRI (*p* = 0.245) between the two groups (Table 2). AARPRI, FIB-4, mFIB-4, FibroQ and Forns index with *p* ≤ 0.1 were included in subsequent logistic regression.

The binary logistic regression Model 1, which adjusted for age, sex, hematoma location, and time from symptom onset to baseline CT scan, showed significant associations between substantial HE and each of the following indices: AARPRI [odds ratio (OR) 1.29, 95% confidence interval (CI) 1.03-1.60], FIB-4 (OR 1.16, 95% CI 1.03-1.30) and mFIB-4 (OR 1.05, 95% CI 1.00-1.10). In contrast, substantial HE was not significantly associated with FibroQ or the Forns index **(**Table 3**).** In Model 2, which additionally considered the baseline hematoma volume, substantial HE was significantly associated only with AARPRI (OR 1.26, 95% CI 1.00-1.57) and FIB-4 (OR 1.15, 95% CI 1.02-1.30).

**Table 3:** Associations between liver fibrosis indices and substantial HE in patients within 24 h of onset (n = 436)

Index	Odds ratio (95% confidence interval); p-value	
	Model 1	Model 2
AARPRI	1.286 (1.032-1.602); p = 0.025	1.256 (1.003-1.573); *p* = 0.047
FIB-4	1.155 (1.026-1.300); *p* = 0.017	1.149 (1.018-1.297); *p* = 0.025
mFIB-4	1.051 (1.001-1.103); p = 0.047	1.045 (0.994-1.098); *p* = 0.084
FibroQ	1.044 (0.999-1.092); *p* = 0.057	1.039 (0.992-1.088); *p* = 0.108
Forns index	1.124 (0.980-1.290); *p* = 0.095	1.127 (0.980-1.295); *p* = 0.093

*Abbreviations*: *AARPRI* AAR/platelet ratio index, where AAR is aspartate aminotransferase/alanine aminotransferase (AST/ALT) ratio, *FIB-4* fibrosis-4, *mFIB-4* modified fibrosis-4, *FibroQ* fibrosis quotient, *ICH* intracerebral hemorrhage, *CI* confidence interval

Model 1 was adjusted for age, sex, hematoma location and time from symptom onset to baseline computed tomography scan

Model 2 was adjusted for the same variables as Model 1, as well as baseline hematoma volume

### Association between liver fibrosis indices and substantial HE in patients within 6 h of ICH onset

Of patients within 6 h of symptom onset (*n* = 200), 60 (30.0%) had substantial HE (Supplementary Table 1). For univariate analysis, the results were similar to those from patients admitted within 24 h of onset, except age (*p* = 0.096).

All six liver fibrosis indices, including APRI, AARPRI, FIB-4, mFIB-4, FibroQ and Forns index were associated with substantial HE in the univariate analysis and included in subsequent logistic regression (all p<0.05, Supplementary Table 1). After adjusting the following variables in the binary logistic regression Model 1: age, sex and hematoma location, all six liver fibrosis indices were associated with substantial HE: APRI (OR 2.63, 95% CI 1.30-5,34), AARPRI (OR 1.53, 95% CI 1.08-2.16), FIB-4 (OR 1.34, 95% CI 1.07-1.67), mFIB-4 (OR 1.08, 95% CI 1.00-1.17), FibroQ (OR 1.08, 95% CI 1.00-1.16) and Forns index (OR 1.37, 95% CI 1.10-1.69). In Model 2, after considering the baseline hematoma volume, substantial HE was still significantly associated with all six liver fibrosis indices **(**all p<0.05, Table 4**)**.

**Table 4:** Associations between liver fibrosis indices and substantial HE in patients within 6 h of onset (*n* = 200)

Index	Odds ratio (95% confidence interval); ***p***-value	
	Model 1	Model 2
APRI	2.634 (1.299-5.341); *p* = 0.007	2.641 (1.303-5.356); p = 0.007
AARPRI	1.526 (1.081-2.156); *p* = 0.016	1.553 (1.093-2.207); *p* = 0.014
FIB-4	1.336 (1.071-1.667); *p* = 0.010	1.345 (1.077-1.679); *p* = 0.009
mFIB-4	1.084 (1.003-1.170); *p* = 0.041	1.088 (1.006-1.177); *p* = 0.034
FibroQ	1.076 (1.001-1.156); *p* = 0.048	1.080 (1.003-1.163); *p* = 0.042
Forns index	1.365 (1.104-1.689); *p* = 0.004	1.366 (1.104-1.689); p = 0.004

*Abbreviations*: *APRI* AST-platelet ratio index, *AARPRI* AAR/platelet ratio index, where AAR is aspartate aminotransferase/alanine aminotransferase (AST/ALT) ratio, *FIB-4* fibrosis-4, *mFIB-4* modified fibrosis-4, *FibroQ* fibrosis quotient, *ICH* intracerebral hemorrhage, *CI* confidence interval

Model 1 was adjusted for age, sex and hematoma location

Model 2 was adjusted for the same variables as Model 1, as well as baseline hematoma volume

## Discussion

In a Chinese population with a largely normal range of liver chemistry, we explored the association between various liver fibrosis indices and substantial HE after primary ICH. We found that at least two single liver fibrosis indices were independently associated with increased risk for substantial HE. Among primary ICH patients within 24 h of symptom onset, AARPRI and FIB-4 were associated with substantial HE. While in patients within 6 h of symptom onset, all six liver fibrosis indices were independently associated with substantial HE.

Growing evidence suggests an association between subclinical liver disease, such as liver fibrosis, and cerebrovascular disease. For example, the presence of liver fibrosis assessed with transient elastography is a strong predictor of long-term, all-cause mortality in the ischemic stroke population as well as a predictor of risk of ischemic stroke [26, 27]. Another study showed liver fibrosis, as measured using FIB-4, to be independently associated with risk of hemorrhagic transformation in acute ischemic stroke patients [28]. Consistent with these findings, our study indicates an association between liver fibrosis indices and substantial HE among ICH patients with largely normal standard liver chemistry. Our findings are consistent with a retrospective analysis of the Virtual International Stroke Trials Archive ICH database linking liver fibrosis (as assessed using two serum-based liver fibrosis indices) to admission hematoma volume, HE and 3-month mortality in Caucasians with normal liver chemistry [22]. In addition, one study reported an association between subclinical alterations in individual hepatic enzymes, such AST and ALP, and worse clinical outcomes among ICH patients [12]. This result was based on univariable analysis, and these associations were no longer significant after adjusting for confounders.

In our study, one in four patients experienced HE after primary ICH, consistent with studies of HE incidence ranging from 13% to 38% in the United States, Japan and Italy [4, 29]. In our analysis, age, sex, hematoma location, baseline hematoma volume and platelet count were significantly associated with HE. Although the mechanisms underlying the association between liver fibrosis and HE remain obscure, some hypotheses can be proposed. First, the liver synthesizes numerous clotting factors involved in the coagulation cascade. Patients with liver disease may cause coagulation factor deficiency, leading to blood clotting abnormalities and increasing risk of developing HE [29]. In addition, progression of liver fibrosis reduces the production of thrombopoietin and, hence, reduces platelet production [30]. Second, vessel wall damage and vascular inflammation are associated with the development of fibrosis [31]. In fact, one study suggested that subclinical coagulopathy, endothelial dysfunction, and vascular inflammation may be possible mechanisms between liver fibrosis and increased risk of HE [22]. Further research should clarify how liver fibrosis affects risk of HE after ICH.

Our study has several limitations. First, it was a retrospective analysis based on data from a single center. Second, hematoma size was calculated using the A*B*C/2 formula, while is widely used [24] but may not be as accurate as semi-automated measurement of hematoma size due to focusing on intraparenchymal HE assessment without IVH considered and not volumetrically measure [32]. Third, liver fibrosis indices may be less accurate in patients without known liver diseases than more invasive, costlier methods such as liver biopsy and advanced imaging.

## Conclusions

Despite the limitations, our study provides evidence that subclinical liver disease, as assessed with liver fibrosis indices, is related to risk of substantial HE after primary ICH in a Chinese cohort. These findings highlight the necessity of taking liver fibrosis into account routinely when screening ICH patients, for risk of substantial HE. Further studies should investigate how liver fibrosis affects HE after ICH.

## Supplementary Information

**Additional file 1: Supplementary Table 1.** Characteristics of ICH patients within 6 h of ICH symptom onset (*n* = 200), stratified by HE.

## Data Availability

The datasets used and analysed during the current study are available from the corresponding author on reasonable request.
