# Supplementary material for: Liver fibrosis indices associated with substantial hematoma expansion in Chinese patients with primary intracerebral hemorrhage
Source: BMC Neurol. 2021 Dec 9;21:478. doi: 10.1186/s12883-021-02494-0 (PMC8656098; doi:10.1186/s12883-021-02494-0)
Supplement: Supplementary file 1 — Additional file 1: Supplementary Table 1. Characteristics of ICH patients within 6 h of ICH symptom onset (n = 200), stratified by HE. [file 12883_2021_2494_MOESM1_ESM.docx]

**Supplementary Table 1.** Characteristics of ICH patients within 6 hours of ICH symptom onset (n=200), stratified by HE

| Characteristic | With HE  (n = 60) | Without HE  (n= 140) | p Value | OR (95% CI) |
| --- | --- | --- | --- | --- |
| Age, years | 63.0 (12.4) | 59.4 (14.4) | 0.096 | NA |
| Male | 49 (81.7) | 93 (66.4) | 0.030 | 2.251 (1.072-4.728) |
| GCS | 14 (8-15) | 14 (9-15) | 0.987 | NA |
| NIHSS | 9 (4-17) | 10 (4-18) | 0.965 | NA |
| BP on admission, mmHg |  |  |  | NA |
| Systolic BP | 169.1 (26.3) | 171.0 (26.7) | 0.634 |  |
| Diastolic BP | 95.5 (16.9) | 98.0 (17.3) | 0.358 |  |
| Comorbidities |  |  |  |  |
| Hypertension | 39 (65.0) | 86 (61.4) | 0.633 | 1.166 (0.621-2.190) |
| Diabetes mellitus | 7 (11.7) | 12 (8.6) | 0.494 | 1.409 (0.526-3.775) |
| Dyslipidemia | 17 (28.3) | 47 (33.6) | 0.467 | 0.782 (0.403-1.517) |
| Heart disease | 5 (8.3) | 11 (7.9) | 1.000 | 1.066 (0.354-3.213) |
| Stroke history | 7 (11.7) | 13 (9.3) | 0.607 | 1.290 (0.488-3.414) |
| Smoking | 21 (35.0) | 36 (25.7) | 0.182 | 1.556 (0.810-2.986) |
| Alcohol consumption | 21 (35.0) | 40 (28.6) | 0.366 | 1.346 (0.706-2.565) |
| HBV infection | 48 (80.0) | 97 (69.3) | 0.120 | 1.773 (0.857-3.670) |
| Antiplatelet drugs | 4 (6.7) | 3 (2.1) | 0.201 | 3.262 (0.707-15.047) |
| ICH volume, ml | 19.7 (7.7-35.3) | 11.0 (6.38-30.0) | 0.047 | NA |
| Location of bleed |  |  | 0.009 | NA |
| Deep location | 36 (60.0) | 109 (77.9) |  |  |
| Lobar location | 18 (30.0) | 17 (12.1) |  |  |
| Infratentorial location | 6 (10.0) | 14 (10.0) |  |  |
| IVH | 10 (16.7) | 38 (27.1) | 0.112 | 0.537 (0.247-1.165) |
| Symptom onset to first CT, h | 3.8 (3.0-5.0) | 4.0 (3.0-5.0) | 0.414 | NA |
| Time between the first and second CT, h | 21.5 (9.1-32.0) | 24.5 (10.3-38.0) | 0.197 | NA |
| Laboratory findings |  |  |  | NA |
| Platelet count, ×10^9^/L | 135 (110-201) | 167 (135-200) | 0.006 |  |
| International normalized ratio | 1.0 (0.9-1.0) | 1.0 (0.9-1.0) | 0.382 |  |
| Prothrombin time, sec | 11.3 (10.6-12.3) | 11.3 (10.7-12.0) | 0.826 |  |
| APTT, sec | 27.3 (24.4-29.6) | 26.2 (24.1-30.0) | 0.420 |  |
| Aspartate transaminase, IU/L | 28 (21-35) | 25 (20-34) | 0.204 |  |
| Alanine transaminase, IU/L | 21 (17-34) | 22 (15-33) | 0.833 |  |
| Albumin, g/dL | 43.1 (41.1-46.1) | 43.6 (41.3-46.5) | 0.394 |  |
| Liver fibrosis index |  |  |  | NA |
| APRI | 0.5 (0.3-1.0) | 0.4 (0.3-0.6) | 0.023 |  |
| AARPRI | 1.2 (0.9-1.9) | 1.1 (0.8-1.6) | 0.037 |  |
| FIB 4 | 2.6 (1.8-4.0) | 2.1 (1.4-2.8) | 0.004 |  |
| mFIB 4 | 5.2 (3.5-8.4) | 4.1 (2.6-7.0) | 0.007 |  |
| FibroQ | 5.3 (3.5-7.9) | 3.8 (2.5-7.0) | 0.019 |  |
| Forns index | 7.0 (5.6-8.4) | 6.1 (4.9-7.2) | 0.003 |  |

Values are n (%), mean (SD) or median (interquartile range).

Abbreviations: APRI, AST to platelet ratio index; AARPRI, AAR/platelet ratio index, where AAR is aspartate aminotransferase/alanine aminotransferase (AST/ALT) ratio; FIB-4, fibrosis-4; mFIB-4, modified fibrosis-4; FibroQ, fibrosis quotient; GCS, Glasgow Coma Scale; NIHSS, National Institutes of Health Stroke Scale; BP, blood pressure; HBV, hepatitis B virus; CT, computed tomography; HE, hematoma expansion; ICH, intracerebral hemorrhage; IVH, intraventricular hemorrhage; APTT, activated partial thromboplastin time.
